# Emotion recognition based on multi-modal physiological signals and transfer learning

**DOI:** 10.3389/fnins.2022.1000716

**Published:** 2022-09-08

**Authors:** Zhongzheng Fu, Boning Zhang, Xinrun He, Yixuan Li, Haoyuan Wang, Jian Huang

**Affiliations:** School of Artificial Intelligence and Automation, Huazhong University of Science and Technology, Wuhan, China

**Keywords:** emotion recognition, transfer learning, domain adaptation, physiological signal, multimodal fusion, individual difference

## Abstract

In emotion recognition based on physiological signals, collecting enough labeled data of a single subject for training is time-consuming and expensive. The physiological signals’ individual differences and the inherent noise will significantly affect emotion recognition accuracy. To overcome the difference in subject physiological signals, we propose a joint probability domain adaptation with the bi-projection matrix algorithm (JPDA-BPM). The bi-projection matrix method fully considers the source and target domain’s different feature distributions. It can better project the source and target domains into the feature space, thereby increasing the algorithm’s performance. We propose a substructure-based joint probability domain adaptation algorithm (SSJPDA) to overcome physiological signals’ noise effect. This method can avoid the shortcomings that the domain level matching is too rough and the sample level matching is susceptible to noise. In order to verify the effectiveness of the proposed transfer learning algorithm in emotion recognition based on physiological signals, we verified it on the database for emotion analysis using physiological signals (DEAP dataset). The experimental results show that the average recognition accuracy of the proposed SSJPDA-BPM algorithm in the multimodal fusion physiological data from the DEAP dataset is 63.6 and 64.4% in valence and arousal, respectively. Compared with joint probability domain adaptation (JPDA), the performance of valence and arousal recognition accuracy increased by 17.6 and 13.4%, respectively.

## Introduction

Emotion is a complex expression that integrates people’s psychological and physiological functions. It reflects the subjective response of individuals to external stimuli all the time (Sharot et al., 2004). Since affective computing was proposed, researchers have devoted to digitizing the concept of emotion, enabling computers to recognize and process it, and providing more reliable signal input for human-computer interaction (Picard, 2003; Mühl et al., 2014). In the human-computer interaction system, accurately decoding the user’s emotion can make the device not only passively receive the user’s instructions but also truly perceive the user’s state, to better understand the user’s intention and establish a more natural and harmonious human-computer interaction environment (Egger et al., 2019). As a research hotspot in human-computer interaction, affective computing is widely used in traffic safety (Liu et al., 2019; Du et al., 2020), brain-computer interface (Al-Nafjan et al., 2017; Rao et al., 2018), medical health (Hosseinifard et al., 2013; Huang et al., 2019), and other fields. Affective computing includes three continuous processes: emotion recognition, behavior generation, and induction. Accurate emotion recognition is the basis for building a good human-computer interaction experience (Egger et al., 2019). However, in practical applications, collecting large numbers of data for each user to train the classifier is difficult, and the recognition accuracy is easily affected by data noise (Wan et al., 2021). When the accuracy of emotion recognition is influenced by physiological signals’ individual differences and inherent noise, making the model trained in the existing data set accurately identify new users’ emotions without collecting data or collecting as little data as possible has essential research value and application significance.

Nowadays, there are many emotion recognition methods, such as analyzing users’ voices (Li et al., 2019; Shaqra et al., 2019), facial expressions (Lawrence et al., 2015; Abdulsalam et al., 2019), and physiological signals (He et al., 2017; Liao et al., 2020). Physiological signals are the most easily acquired signals by the human body through sensors. It contains many important physiological and psychological information about the human body and plays a significant role in computer recognition of human emotions (Li et al., 2021). Compared with emotion recognition based on facial expression, emotion recognition based on physiological signals not only has the advantages of low cost and high efficiency in data acquisition but also can avoid the errors caused by light and shadow acquisition and the invasion of user privacy (Hao et al., 2020; Fu et al., 2021).

In the aspect of emotion recognition, electroencephalogram (EEG) has been paid more attention by researchers among many physiological signals. The analysis of EEG signals in the field of emotion recognition depends on data preprocessing, feature extraction, and feature classification (Xie et al., 2021). Many researchers use traditional machine learning or deep neural network to classify EEG signals by extracting the energy features of the delta, theta, alpha, beta, and gamma bands. For example, Verma and Tiwary (2014) extracted the relative power energy, logarithmic relative power energy, absolute logarithmic relative power energy, standard deviation, and spectral entropy features of five frequency bands from EEG signals. Liu et al. (2016) used a deep autoencoder to extract the features of EEG signals in the DEAP dataset and extract features. Sorkhabi (2014) used continuous wavelet transform to extract energy features of five frequency bands and entropy features of wavelet coefficients. Yin et al. (2017) extracted the frequency band power features, statistical features, signal zero crossing rate, Shannon entropy, spectral entropy, kurtosis, skewness, and other features of the five frequency bands. Torres-Valencia et al. (2017) extracted statistical features of EEG signals and power features of five frequency bands.

However, a single EEG signal’s lack of feature information will lead to low emotion recognition accuracy. Some researchers use feature level fusion or signal level fusion to fuse multimodal signals to improve emotion recognition accuracy. He et al. (2021) extracted 11 features from the EEG signal of FP2 channel and 6 features from HR. Using multi-core learning for fusion, they achieved 67% binary classification accuracy under fewer signals and channels. Song et al. (2019) used attention-based long-term short-term memory to fuse multimodal physiological signals, including electroencephalogram (EEG), Galvanic Skin Response (GSR), respiration (RSP), and electrocardiogram (ECG), to improve the classification accuracy. Our study uses EEG, RSP, GSR, and photo-plethysmograph (PPG) signals collected from the database for emotion analysis using physiological signals (DEAP) (Koelstra et al., 2011) for feature extraction. We concatenate four mode features to achieve multimodal feature fusion. It can remedy the inherent limitations of the single mode by providing more dimensional features. Consequently, the multimodal features improve the accuracy of emotion recognition.

The EEG signals are very complex due to the inherent non-stationary, non-linear, and non-Gaussian characteristics (Subha et al., 2010). Meanwhile, EEG signals are greatly affected by age, psychology, and other factors, which result in significant differences in individual EEG signals (Lotte et al., 2018). This difference is often substantial and cannot be ignored. The traditional emotion recognition based on EEG does not consider the existence of differences and directly trains a general model. The difference between EEG signals of different individuals will directly affect the accuracy of model recognition and classification and lead to a poor generalization ability of the model (Zheng and Lu, 2016). Considering different types of information in EEG signals make it difficult to filter out information sensitive to specific tasks, and there are few similar EEG data among different individuals due to the significant difference in EEG, it is problematic that use the deep learning model based on old user data training to estimate the mental state of new users (Wan et al., 2021).

In order to solve the above problems, some researchers have introduced transfer learning to emotion recognition. Li et al. (2019) proposed a multi-source transfer learning algorithm to transfer the existing emotion model to new subjects. The experimental results show that this method can effectively reduce the demand for data quantity and increase the calibration capability of the model. Chai et al. (2017) proposed an adaptive subspace feature matching algorithm for emotion recognition, which aligns the source and target subspaces by learning linear transformation to reduce the distribution discrepancy between the source and target domains. Lin and Jung (2017) proposed a conditional transfer learning framework. The algorithm first evaluates the individual’s transferability to positive transfer and then selectively leverages the data from others with comparable feature spaces. Therefore, in order to solve the low accuracy of emotion recognition caused by the mismatch between individual specificity and global threshold, we introduce domain adaptation, a transfer learning method, into emotion recognition. This method can apply the patterns learned in one domain to other domains and reduce the differences in EEG data distribution so that to improve the model’s ability to recognize new users’ emotions.

Generally, the domain adaptation method usually seeks the alignment between the source and target domains. Different domain adaptation methods often use different alignments. The current alignment methods can be divided into three categories according to distribution matching schemes: domain-level, class-level, and sample-level (Lu et al., 2021). Pan et al. (2010) proposed the transfer component analysis (TCA) method, which uses the maximum mean difference (MMD) to learn a transformation matrix in the reproducing kernel Hilbert space (RKHS) to align the marginal distribution between the two domains. Long et al. (2017) proposed the joint distribution adaptation (JDA) method to align the joint distribution of multiple domains through multi-kernel MMD. Sun et al. (2017) proposed the correlation alignment (CORAL) method, which minimizes the domain shift by aligning the second-order statistical data of source and target distribution. The above commonly used domain adaptation methods belong to domain-level matching. The domain-level matching completely ignores the intra-domain data structure. It is too rough to miss some details and challenging to achieve good matching results.

The sample level matching can avoid the problem that domain-level matching ignores intra-domain data structure. Courty et al. (2017) proposed a regularized unsupervised optimal transport model, which uses the optimal transport theory to calculate the distance between the probability distributions of the source and the target domain. In the research of Das and Lee (2018), the source and the target domain are regarded as hypergraphs, and the first-order, second-order, and third-order similarities between graphs are used for class-regularized hypergraph matching to obtain the matching between the samples of the source domain and the target domain. However, sample level matching is very time-consuming, and it is more prone to overfitting when local information is affected by the noise.

Class-level matching can neutralize too rough domain-level matching and too fine sample-level matching. Wang et al. (2018) proposed the Stratified Transfer Learning (STL) method. STL transforms the same classes in the source and the target domain into the same subspace and uses the intra-affinity of the class to perform knowledge migration within the class. Tian et al. (2020) proposed the Centroid Matching and local Manifold Self-learning (CMMS) method. CMMS can thoroughly explore the data distribution structure of the domain and minimize the distribution difference in domain adaptation by combining class centroid matching with local manifold self-learning. Lu et al. (2021) proposed a domain adaptation method based on substructure level matching, which regards a class as synthesizing multiple substructures and aligning the substructures. The above commonly used domain adaptation methods belong to class-level matching. Considering that the EEG signal acquisition process contains the location information of different channels, which has the intra-domain data structure, we adopt the class-level domain adaptation to avoid rough alignment of domain-level adaptation and overfitting of sample-level adaptation.

In the matching process of the source and target domains, it is necessary to project the source and target domains into the same feature space through the projection matrix. The TCA, JDA, BDA, and JPDA all uses the single projection matrix for transfer (Pan et al., 2010; Long et al., 2017; Wang et al., 2017; Zhang et al., 2020). However, the distribution of the source domain and target domain is different, and a single projection matrix cannot account for all the feature distribution of the source and target domains. Therefore, we propose a bi-projection matrix (BPM) to better project the source and target domains into the feature space.

This paper uses EEG, RES, PPG, and GSR signals collected from the DEAP dataset to extract features, and we concatenate four mode features to achieve multimodal feature fusion. Multimodal fusion gives full play to the advantages of each mode and makes up for its inherent limitations, improving the accuracy of emotion recognition. In order to improve the generalization ability of the model, we propose a joint probability domain adaptation method based on the substructure. Substructure-level data is aligned by discriminative joint probability maximum mean discrepancy (DJP-MMD) (Zhang et al., 2020). Substructure-based joint probability domain adaptation (SSJPDA) can avoid inadaptability caused by rough matching and overfitting when learning local information caused by noise points. In order to better project the source and target domains, we propose a method of the bi-projection matrix (BMP), which can effectively avoid data loss in the projection stage.

The main contributions of this study are as follows:

We proposed a substructure-based joint probability domain adaptation method (SSJPDA).
We proposed the bi-projection matrix (BPM) method and applied it to the SSJPDA algorithm.
We validated the SSJPDA algorithm and the SSJPDA-BPM method on DEAP dataset.

The rest of this paper is arranged as follows: Section “Materials and methods” introduces the SSJPDA with the BPM algorithm. Section “Results” presents the results verified on the DEAP dataset. Section “Discussion” gives the full discussion above the result.

## Materials and methods

### Physiological signal dataset

This study adopted the DEAP dataset to inspect our proposed algorithm. DEAP dataset was established by Koelstra et al. (2011) in 2012 and contained 32 subjects. Every subject watched the 40 selected music videos, and each video viewed by the subjects was regarded as an independent experiment. After the video viewing, the subjects need to use the self-evaluation model to score arousal, valence, like/dislike, dominance, and familiarity, providing label information for each signal. Every experiment recorded 40 physiological signals of subjects, of which the first 32 signals were EEG signals collected according to the international 10–20 system, and the remaining 8 signals were peripheral physiological signals, including 2 ophthalmic signals, 1 skin electrical signal, 2 EMG signals, 1 respiratory record, 1 plethysmography, and 1 temperature record. The dataset also preprocessed the collected signals. Each test section’s EEG data and other peripheral physiological signal data were divided into 3 s baseline data and 60 s test data. EEG signals are collected according to the international 10–20 lead system and down-sampling from 512 Hz original sampling frequency to 128 Hz. RES, PPG, and GSR signals are down-sampled to 128 Hz. A band-pass frequency filter of 4–45 Hz and a blind source separation technique were used to remove the eye artifacts.

### Feature extraction

Considering that the subjects are not always in a high emotional activation state if the sliding window is used to divide the data into small segments, many segments will contain useless information (Piho and Tjahjadi, 2018). Therefore, we directly extract features from the preprocessed 60 s experimental data to make samples instead of dividing continuous data into multiple segments and making each segment into samples in the feature processing. We extract the differential entropy features of five frequency bands from each recorded EEG data from each EEG channel. These five frequency bands are related to people’s state of mind, so they also contain information about the state of specific thinking tasks. These five bands are Delta (1–4 Hz), Theta (4–8 Hz), Alpha (8–13 Hz), Beta (13–30 Hz), and Gamma (30–48 Hz). Some studies have shown that the differential entropy feature is superior to the power spectral density feature (PSD) in EEG-based emotion recognition (Zheng and Lu, 2015; Soleymani et al., 2017).

We extract their time-domain and frequency-domain features for the peripheral physiological signals PPG, GSR, and RES. The extracted time-domain features and frequency-domain features refer to numerous previous studies (Verma and Tiwary, 2014; Yin et al., 2017; Zhang et al., 2021). Time-domain features depend on statistical features, which are simple and intuitive. It realizes classification by analyzing statistical features such as mean, maximum, minimum, root mean square, standard deviation, etc. The time-domain analysis contains all the characteristics of physiological signals, and the signal is processed directly. Hence the loss of information is relatively small. For example, from the time domain characteristics of PPG signals, we can analyze the heart rate and its changes, which are closely related to emotional arousal. In addition, Frequency domain features can show the frequency information that time-domain features cannot reach in more detail. Consequently, we got 1,280 samples (32 subjects × 40 samples). Table 1 lists the features extracted from the data.

**TABLE 1:** The features used in Experiment 1 and Experiment 2.

Signal	Feature	Description	Dimension
EEG	Differential Entropy (DE)	DE in different bands: Delta (1–4 Hz), Theta (4–8 Hz), Alpha (8–13 Hz), Beta (13–30 Hz), and Gamma (30–48 Hz)	32 channels × 5 features
PPG	Time Domain	Mean value, maximum value, minimum value, standard deviation and root mean square value of heart rate interval. Heart rate (times/second)	1 channel × 8 features
	Frequency domain	Power spectral density of bands 0.1–1.5 Hz and 1.5–3 Hz.	
GSR	Time Domain	Mean, standard deviation	1 channel × 7 features
	Frequency domain	Power spectral density of bands 0.4–0.8 Hz, 0.8–1.2 Hz, 1.2–1.6 Hz, 1.6–2.0 Hz, and 2.0–2.4 Hz.	
RES	Time Domain	Mean, maximum, minimum, standard deviation, and root mean square value of respiratory interval. Respiratory rate (times / second)	1 channel × 8 features
	Frequency domain	Power spectral density of bands 0.1–1.5 Hz and 1.5–3.0 Hz.	

### The generation of substructures

The source domain $\left\{X_{S},Y_{S}\right\}={\left\{\left({\text{x}}_{s,i},y_{s,i}\right)\right\}}_{i=1}^{n_{s}}$ containing the label recorded as *𝒟_s_*. The target domain $X_{t}={\left\{{\text{x}}_{t,j}\right\}}_{j=1}^{n_{t}}$ without label recorded as *𝒟_t_*. The *n*_*s*_ and *n*_*t*_ are the number of source domain samples and target samples, respectively. x ∈ ℝ*^d×1^* is the feature vector, and *y* ∈ {1, … , *C*} is its label in the *C*-class classification problem. *𝒟_s_* and *𝒟_t_* have the same feature space and label space, but the feature distribution is different, i.e., *P*(*X*_*s*_, *Y*_*s*_) ≠ *P*(*X*_*t*_, *Y*_*t*_). The task of domain adaptation is to reduce the distribution difference between the source domain and the target domain, so as to predict the label *y*_*t*_ of the target domain *𝒟_t_* with the help of the source domain *𝒟_s_* (Lu et al., 2021).

We use δ ∼ *𝒩* (0; σ^2^) and X to represent all feature data and the Gaussian mixture model (GMM) to fit them. The kth component in GMM is recorded as *X*_*k*_ ∼ *𝒩* (*z*_*k*_, σ_*k*_) where *z*_*k*_ represents mean value and σ_*k*_ represents covariance. Our goal is to get mean value *z*_*k*_ and covariance σ_*k*_. These GMM parameters can be obtained using the Expectation Maximum (EM) algorithm. Suppose *K*_*s*_ and *K*_*t*_ are the number of GMM components in the source domain and the target domain, respectively. *K*_*s*_ is determined by the Bayesian Information Criterion (BIC), and *K*_*t*_ is manually set according to the specific data set.

After obtaining the GMM of the source domain and the target domain, we regard each component of the GMM as a substructure in the feature space, and the information of the cluster center represents the substructure. Specifically, set


(1) $${\text{μ}}_{s}=\sum_{i=1}^{k_{s}}w_{s,i}{\text{δ}}_{z_{s,i}}$$

(2) $${\text{μ}}_{t}=\sum_{i=1}^{k_{t}}w_{t,i}{\text{δ}}_{z_{t,i}}$$

where μ_*s*_ and μ_*t*_are the distribution of source domain and target domain, respectively. z ∈ ℝ*^d×1^* is cluster center, and δ_*z*_ is the Dirac function at location z . *w*is the probability weight associated with z, where $\sum_{i=1}^{k_{s}}w_{s,i}=1$ and $\sum_{i=1}^{k_{t}}w_{t,i}=1$.

The cost between z_*i*_ and z_*j*_ in square Euclidean distance can be expressed as


(3) $$c\left(z_{s,i},z_{t,j}\right)={||z_{s,i}-z_{t,j}||}_{2}^{2}$$

Therefore, the problem can be regarded as the partial optimal transmission (POT) problem, and the upper bound *w*_*s,i*_ is 1. The total cost of POT is ⟨π, *C*⟩*_F_* that is the Frobenius dot product of cost matrix *C* and coupling matrix π. The *C* ∈ ℝ*^k_s_×k_t_^* represents the cost of μ_*s*_ and μ_*t*_ distribution, and the π ∈ ℝ*^k_s_×k_t_^* represents the coupling between μ_*s*_ and μ_*t*_distribution.

The goal is to obtain the optimal transmission, which can be expressed as


(4) $$\begin{array}{c} \pi_{1}^{*}=\arg\min_{\pi }{\left\langle \pi ,C\right\rangle }_{F}+\lambda_{1}H\left(\pi \right)\\ \text{s}.t.\pi^{T}1_{k_{s}}=w_{t} \end{array}$$

where $H\left(\pi \right)=\sum_{ij}\pi_{ij}\log\pi_{ij}$ is the entropy term, and λ_*1*_ is the super parameter to balance the speed and accuracy calculation.

The feasible solution set of π*^T^*1*_k_s__* = *w*_*t*_ is *C*_1_, and then it can be solved by the Lagrange method. Thus, we can easily get the optimal π*.


(5) $$\pi_{1}^{*}={\text{π}}_{0}diag\left(w_{t}\text{⊘}{\text{π}}_{0}^{T}1_{k_{s}}\right)$$

where ${\text{π}}_{0}=\exp\left(-\frac{\text{C}}{\lambda_{1}}-1\right)$ and ⊘ represent element-wise divide and *diag*represents the diagonals. Once the coupling matrix ${\text{π}}_{1}^{*}$ is obtained, the source domain weights can be easily calculated as $w_{s}={\text{π}}_{1}^{*}1_{k_{t}}$.

### Substructural joint probability domain adaptation

The domain adaptation (DA) method attempts to find a mapping *h*. The source domain and target domain are mapped to the same subspace, so that the classifier trained on *h*(*x*_*s*_)can achieve good classification effect on *h*(x_*t*_). For example, a linear map*h*(x) = *A*^T^xfor the source and the target domains, where *A* ∈ ℝ*^d× p^*, *p* ≤ *d*.

Due to the difference between the source domain and the target domain, it is generally assumed that their probabilities distributions are not equal. The derivation of TCA, JDA and BDA algorithms are based on the inequality of the marginal probabilities *P*(*X*_*s*_) ≠ *P*(*X*_*t*_) or the conditional probabilities *P*(*Y*_*s*_|*X*_*s*_) ≠ *P*(*Y*_*t*_|*X*_*t*_). However, the JPDA algorithm derives from the inequality assumption of joint probabilities *P*(*X*_*s*_, *Y*_*s*_) ≠ *P*(*X*_*t*_, *Y*_*t*_). Because JPDA directly considers the difference of joint probability distribution, the performance of JPDA is better than the traditional DA method, which JPDA can improve the between-domain transferability and the between-class discrimination (Zhang et al., 2020).

After obtaining the substructure, the set of substructures in source domain is recorded as $\left\{Z_{S},Y_{S}^{\prime }\right\}={\left\{\left({\text{z}}_{s,i},y_{s,i}^{\prime }\right)\right\}}_{i=1}^{k_{s}}$, and the set of substructures in target domain is recorded as $Z_{t}={\left\{z_{t,j}\right\}}_{j=1}^{k_{t}}$, where *k*_*s*_ and *k*_*t*_ are the number of source domain substructure and target domain substructure, respectively.

Let the source domain substructure one-hot coding label matrix be $Y_{s}^{{}^{\prime }}=\left[{\text{y}}_{s,1}^{\prime };\ldots ;{\text{y}}_{s,k_{\text{s}}}^{\prime }\right]$ and the predicted target domain substructure one-hot coding label matrix be ${\hat{Y}}_{t}^{\prime }=\left[{\hat{\text{y}}}_{t,1}^{\prime };\ldots ;{\hat{\text{y}}}_{t,k_{t}}^{\prime }\right]$ where ${\text{y}}_{s,ks}^{\prime }\in {\mathbb{R}}^{1\times C}$ and ${\hat{\text{y}}}_{t,kt}^{\prime }\in {\mathbb{R}}^{1\times C}$. Define


(6) $$F_{s}=\left[Y_{s}^{\prime }\left(:,1\right)*\left(C-1\right),\ldots ,Y_{s}^{\prime }\left(:,C\right)*\left(C-1\right)\right]$$

(7) $${\hat{F}}_{t}=\left[{\hat{Y}}_{t}^{\prime }{\left(:,1:C\right)}_{\hat{c}\neq 1},\ldots ,{\hat{Y}}_{t}^{\prime }{\left(:,1:C\right)}_{\hat{c}\neq C}\right]$$

where $Y_{s}^{\prime }\left(:,c\right)$ denotes the *c*-th column of $Y_{s}^{\prime }$, $Y_{s}^{\prime }\left(:,c\right)*\left(C-1\right)$ repeats $Y_{s}^{\prime }\left(:,C\right)$ *C*−1 times to form a matrix in ℝ^*k_s_*×(*C*−1)^, and ${\hat{Y}}_{t}^{\prime }{\left(:,1:C\right)}_{\hat{c}\neq \text{c}}$ is formed by the 1st to the *C*-th, (except the *c*-th) columns of $Y_{t}^{\prime }$. Clearly, *F*_*s*_ ∈ ℝ^k_*s*_×(*C*(*C*−1))^ and ${\hat{F}}_{t}\in {\mathbb{R}}^{k_{t}\times \left(C\left(C-1\right)\right)}$. *F*_*s*_ is fixed, and ${\hat{F}}_{t}$ is constructed from the pseudo labels, which are updated iteratively.

Therefore, the objective function of JPDA can be written as follows:


(8) $$\begin{array}{c} \min_{\text{A}}{||A^{\text{T}}Z_{s}N_{s}-A^{\text{T}}Z_{t}N_{t}||}_{F}^{2}-\text{μ}{||A^{\text{T}}Z_{s}M_{s}-A^{\text{T}}Z_{t}M_{t}||}_{F}^{2}+\lambda {||A||}_{F}^{2}\\ \text{s}.t.A^{\text{T}}ZHZ^{\text{T}}A=I \end{array}$$

where μ > 0 is a trade-off parameter and λ is a regularization parameter. *N*_*s*_, *N*_*t*_, *M*_*s*_ and *M*_*t*_ are defined as


(9) $$N_{s}=\frac{Y_{s}^{\prime }}{k_{s}},N_{t}=\frac{{\hat{Y}}_{t}^{\prime }}{k_{t}}$$

(10) $$M_{s}=\frac{F_{s}}{k_{s}},M_{t}=\frac{{\hat{F}}_{t}}{k_{t}}$$

where *H* = *I*−1_*k*_ is the centering matrix, in which *k* = *k*_*s*_ + *k*_*t*_ and 1*_k_* ∈ ℝ*^k×k^* is a matrix with all elements being $\frac{1}{k}$.

Let *Z* = [*Z*_*s*_, *Z*_*t*_], then we reach the Lagrange function of Eq. 8


(11) $$𝒥=\text{tr}\left(A^{\text{T}}\left(Z\left(R_{\min}-\text{μ}R_{\max}\right)Z^{\text{T}}+\lambda I\right)A\right)+\mathrm{tr}\left(\eta \left(I-A^{\text{T}}ZHZ^{\text{T}}A\right)\right)$$

where η is Lagrange multiplier, and


(12) $$R_{\min}=\left[\begin{array}{cc} N_{s}N_{s}^{\text{T}} & -N_{s}N_{t}^{\text{T}}\\ -N_{t}N_{s}^{\text{T}} & N_{t}N_{t}^{\text{T}} \end{array}\right]$$

(13) $$R_{\max}=\left[\begin{array}{cc} M_{s}M_{s}^{\text{T}} & -M_{s}M_{t}^{\text{T}}\\ -M_{t}M_{s}^{\text{T}} & M_{t}M_{t}^{\text{T}} \end{array}\right]$$

*R*_max_ and *R*_min_ have dimensionality *k* × *k*.

By setting the derivative ∇*_A_𝒥* = 0, Eq. 17 becomes a generalized eigen-decomposition problem:


(14) $$\left(Z\left(R_{\min}-\text{μ}R_{\max}\right)Z^{\text{T}}+\lambda I\right)A=\eta ZHZ^{\text{T}}A$$

*A* is then formed by the p trailing eigen-vectors. A classifier can then be trained on*A*^T^*Z*_*s*_ and applied to *A*^T^*Z*_*t*_.

The pseudocode of SSJPDA for classification is summarized in Algorithm 1.

**Algorithm 1:** Substructural Joint Probability Distribution Adaptation (SSJPDA)

**Input:** *X*_*S*_ and *X*_*t*_, source and target domain feature matrices; *Y*_*S*_, source domain one-hot coding label matrix; μ, trade-off parameter; λ, regularization parameter; *T*, number of iterations; **Output:** ${\hat{Y}}_{t}$, estimated target domain labels. **Begin:** Use EM for GMM, cluster each class data in the source to obtain $\left\{Z_{S},Y_{S}^{\prime }\right\}={\left\{\left({\text{z}}_{s,i},y_{s,i}^{\prime }\right)\right\}}_{i=1}^{k_{s}}$, and cluster the unlabeled data in target domain to obtain $Z_{t}={\left\{z_{t,j}\right\}}_{j=1}^{k_{t}}$; Compute cost matrix *C* and coupling matrix π using Eq. 3 and Eq. 4, respectively; Compute the weights of source substructures $w_{s}={\text{π}}_{1}^{*}1_{k_{t}}$ and target substructures $w_{t}=\frac{1_{k_{t}}}{k_{t}}$ **for *n* = 1, …, T do** Construct the joint probability matrix *R*_min_ and *R*_max_ by Eq. 12 and Eq. 13; Solve the generalized eigen-decomposition problem in Eq. 14 and select the *p* trailing eigenvectors to construct the projection matrix A; Train a classifier *f* on $\left(A^{\text{T}}{\text{Z}}_{s},Y_{S}^{\prime }\right)$ and apply it to *A*^T^Z_*t*_ to obtain ${\hat{Y}}_{t}^{\prime }={\left\{y_{t,j}^{\prime }\right\}}_{j=1}^{k_{t}}$ which is the label matrix of substructure in target domain $Z_{t}={\left\{z_{t,j}\right\}}_{j=1}^{k_{t}}$ **End for** For each substructure *z*_*t,j*_, assign its label $y_{t,j}^{\prime }$ to all samples it contains, and gets ${\hat{Y}}_{t}={\left\{y_{t,j}\right\}}_{j=1}^{n_{t}}$ **End**

### Substructure-based joint probability domain adaptation algorithm with bi-projection matrix

As described in the previous subsection, the source and target domains have different probability distributions, so applying only a single projection matrix to both domains simultaneously may lack the ability to align their probability distributions well. It is better to make the source domain and the target domain have their own projection matrix to accomplish the distribution alignment task together. On this basis, we take SSJPDA algorithm as an example to explain how to design the projection matrix of source domain and target domain, respectively, and call it SSJPDA-BPM.

Donate the projection matrices of the source domain and the target domain as *A*_*s*_ and *A*_*t*_, respectively. Therefore, the objective function of SSJPDA-BPM can be written as follows:


(15) $$\begin{array}{c} \min_{\text{A}}{||A_{s}^{\text{T}}Z_{s}N_{s}-A_{t}^{\text{T}}Z_{t}N_{t}||}_{F}^{2}-\text{μ}{||A_{s}^{\text{T}}Z_{s}M_{s}-A_{t}^{\text{T}}Z_{t}M_{t}||}_{F}^{2}\\ +\lambda \left({||A_{s}||}_{F}^{2}+{||A_{t}||}_{F}^{2}\right)\\ \text{s}.t.A_{s}^{\text{T}}Z_{s}H_{s}Z_{s}^{\text{T}}A_{s}=I_{k_{s}},A_{t}^{\text{T}}Z_{t}H_{t}Z_{t}^{\text{T}}A_{t}=I_{k_{t}} \end{array}$$

where *H*_*s*_ = *I_k_s__* − 1*_k_s__* (or *H*_*t*_ = *I_k_t__* − 1_*k_t_*_) is the centering matrix, in which 1_*k_s_*_ ∈ ℝ*^k_s_×k_s_^* (or 1_*k_t_*_ ∈ ℝ*^k_t_×k_t_^*) is a matrix with all elements being $\frac{1}{k_{s}}$ (or $\frac{1}{k_{t}}$).

Let $Z_{A}=\left[A_{s}^{\text{T}}Z_{s},A_{t}^{\text{T}}Z_{t}\right]$, then we reach the Lagrange function of Eq. 15


$$𝒥=tr\left(Z_{A}RZ_{A}^{\text{T}}\right)+tr\left(\eta_{s}\left(I_{k_{s}}-A_{s}^{\text{T}}Z_{s}H_{s}Z_{s}^{\text{T}}A_{s}\right)\right)$$

(16) $$+tr\left(\eta_{t}\left(I_{k_{t}}-A_{t}^{\text{T}}Z_{t}H_{t}Z_{t}^{\text{T}}A_{t}\right)\right)+tr\left(A_{s}^{\text{T}}A_{s}\right)+tr\left(A_{t}^{\text{T}}A_{t}\right)$$

where η_*s*_ η_*t*_ are Lagrange multipliers, and


$$R=R_{\min}-\text{μ}R_{\max}=\left[\begin{array}{cc} R_{11}R_{12} & \\ R_{21}R_{22} & \end{array}\right]$$

(17) $$=\left[\begin{array}{cc} \;N_{s}N_{s}^{\text{T}}-\text{μ}M_{s}M_{s}^{\text{T}}\;-N_{s}N_{t}^{\text{T}}+\text{μ}M_{s}M_{t}^{\text{T}} & \\ -N_{t}N_{s}^{\text{T}}+\text{μ}M_{t}M_{s}^{\text{T}\;}N_{t}N_{t}^{\text{T}}-\text{μ}M_{t}M_{t}^{\text{T}} & \end{array}\right]$$

By setting the derivative ∇_*A_s_*_*𝒥* = 0, ∇_*A_s_*_*𝒥* = 0, and add a constraint $Z_{s}R_{12}Z_{t}^{T}A_{s}=Z_{t}R_{21}Z_{s}^{T}A_{t}$, then Eq. 16 becomes two generalized eigen-decomposition problem:


(18) $$\left(Z_{s}R_{11}Z_{s}^{\text{T}}+Z_{t}R_{21}Z_{s}^{\text{T}}+\lambda I\right)A_{s}=\eta_{s}Z_{s}H_{s}Z_{s}^{\text{T}}A_{s}$$

(19) $$\left(Z_{t}R_{22}Z_{t}^{\text{T}}+Z_{s}R_{12}Z_{t}^{\text{T}}+\lambda I\right)A_{t}=\eta_{t}Z_{t}H_{t}Z_{t}^{\text{T}}A_{t}$$

*A*_*s*_ and *A*_*t*_ are then formed by the p trailing eigen-vectors of each problem. A classifier can then be trained on $A_{s}^{\text{T}}Z_{s}$ and applied to $A_{t}^{\text{T}}Z_{t}$.

The pseudocode of SSJPDA-BPM for classification is summarized in Algorithm 2.

**Algorithm 2:** Substructural Joint Probability Distribution Adaptation with Bi-Projection Metrix (SSJPDA-BPM)

**Input:** *X*_*S*_ and *X*_*t*_, source and target domain feature matrices; *Y*_*S*_, source domain one-hot coding label matrix; μ, trade-off parameter; λ, regularization parameter; *T*, number of iterations; **Output:** ${\hat{Y}}_{t}$, estimated target domain labels. **Begin:** Use EM for GMM, cluster each class data in the source to obtain $\left\{Z_{S},Y_{S}^{\prime }\right\}={\left\{\left({\text{z}}_{s,i},y_{s,i}^{\prime }\right)\right\}}_{i=1}^{k_{s}}$, and cluster the unlabeled data in target domain to obtain $Z_{t}={\left\{z_{t,j}\right\}}_{j=1}^{k_{t}}$; Compute cost matrix *C* and coupling matrix π using Eq. 3 and Eq. 4 respectively; Compute the weights of source substructures $w_{s}={\text{π}}_{1}^{*}1_{k_{t}}$ and target substructures $w_{t}=\frac{1_{k_{t}}}{k_{t}}$ **for *n* = 1, …, T do** Construct the joint probability matrix *R* in Eq. 17 Solve the generalized eigen-decomposition problem in Eq. 18 and Eq. 19, and select the *p* trailing eigenvectors to construct the projection matrix *A*_*s*_ and *A*_*t*_; Train a classifier *f* on $A_{s}^{\text{T}}Z_{s}$ and applied to $A_{t}^{\text{T}}Z_{t}$ to obtain ${\hat{Y}}_{t}^{\prime }={\left\{y_{t,j}^{\prime }\right\}}_{j=1}^{k_{t}}$ which is the label matrix of substructure in target domain $Z_{t}={\left\{z_{t,j}\right\}}_{j=1}^{k_{t}}$ **End for** For each substructure *z*_*t,j*_, assign its label $y_{t,j}^{\prime }$ to all samples it contains, and gets ${\hat{Y}}_{t}={\left\{y_{t,j}\right\}}_{j=1}^{n_{t}}$ **End**

### Validation of the substructure-based joint probability domain adaptation algorithm and substructural joint probability distribution adaptation with bi-projection metrix

The DEAP dataset contains 32 subjects, each taking turns as the target domain and the remaining 31 people as the source domain. The number of samples in the source domain is 1,240 (31 subjects × 40 samples), and the number of target domain samples is 40 (1 subject × 40 samples). After dividing the source and target domains, the EEG, GSR, PPG, and RES modes were transferred, respectively, and all the subjects’ valence and arousal dimensions were classified, respectively. In each sample, the feature dimension of EEG is 160, the feature dimension of GSR is 7, the feature dimension of PPG is 8, and the feature dimension of RES is 8. Those four modes were fused through average splicing, where the feature dimension after fusion in each sample is 183. The feature dimension of the modes remains the same dimension before and after the transfer learning. The effects of single-mode transfer and multi-mode transfer are compared to explore whether data fusion can promote the accuracy of the transfer learning algorithm. By comparing SSJPDA with other transfer learning methods and traditional machine learning methods, this paper explores whether SSJPDA can improve recognition accuracy.

Hyperparameters of the model will affect the recognition accuracy. We divide the target domain with 40 samples from 1 subject into a verification set and a test set for the specific hyperparameter configuration in the algorithm, which follows similar protocols used in Courty et al. (2016). Among them, the training set is an optional 10 samples, and the test set is the remaining 30 samples. Both validation and test sets have no labels. The validation set data and source domain data are trained together to obtain the best accuracy within the range of hyperparameters, and the range of hyperparameter sets follows (Kerdoncuff et al., 2021). Under the best hyperparameters set, the classification accuracy and F1 measure are used to measure the performance of our proposed algorithm on the test set.

## Result

### Experiment 1

In Experiment 1, JPDA, JPDA (BMP), SSJPDA, and SSJPDA (BMP) algorithms were used to transfer EEG, PPG, GSR, RES, and four-mode fusion data (ALL) of subjects, respectively. Table 2 shows the average accuracy and F1-measure of 32 subjects in valence and arousal.

**TABLE 2:** The average accuracy (ACC_100%) and F1-measure in different algorithms with single-mode and multi-mode data in valence and arousal classification.

Method	Modality	Valence		Arousal	
		ACC	F1-measure	ACC	F1-measure
JPDA	EEG	0.529	0.563	0.549	0.615
	PPG	0.561	0.603	0.551	0.589
	GSR	0.537	0.578	0.567	0.619
	RES	0.531	0.574	0.509	0.567
	ALL	0.541	0.576	0.568	0.626
JPDA-BPM	EEG	0.536	0.605	0.525	0.624
	PPG	0.536	0.63	0.551	0.582
	GSR	0.553	0.446	0.537	0.57
	RES	0.517	0.555	0.537	0.613
	ALL	0.533	0.615	0.573	0.613
SSJPDA	EEG	0.604	0.617	0.614	0.645
	PPG	0.588	0.537	0.633	0.634
	GSR	0.605	0.596	0.613	0.618
	RES	0.614	0.643	0.619	0.614
	ALL	0.617	0.627	0.635	0.643
SSJPDA-BPM	EEG	0.621	0.645	0.629	0.655
	PPG	0.62	0.619	**0.648**	0.652
	GSR	0.608	0.581	0.62	0.65
	RES	0.595	0.601	0.636	0.653
	ALL	**0.636**	**0.653**	0.644	**0.679**

The numbers in bold indicate the highest value of the experimental results.

Table 2 shows that in the DEAP dataset, the recognition accuracy of multimodal fusion data is less improved than that of single-mode data recognition. Even in the identification of some modes of JPDA and JPDA-BPM, the accuracy of single-mode is higher than that of multi-mode. However, this phenomenon does not appear in the domain adaptation algorithm using substructure. In the classification of valence and arousal by SSJPDA and SSJPDA-BPM algorithms, the recognition accuracy and F1-measure based on multimodal data are generally higher than that of single-mode data. In the recognition of multimodal data, the recognition accuracy of SSJPDA and SSJPDA-BPM in valence is 14.1 and 19.3% higher than that of JPDA and JPDA-BPM, respectively. In the recognition accuracy of arousal, SSJPDA and SSJPDA-BPM are higher than JPDA and JPDA-BPM by 11.8 and 12.4%, respectively. In the single-mode recognition, SSJPDA-BMP has higher recognition accuracy and F1 than JPDA-BMP in every single mode. Similar rules also appear in the comparison between SSJPDA and JPDA. By comparing the recognition ability of the two transfer learning algorithms with or without the BPM algorithm in each mode, we find that the BPM algorithm is more effective in the transfer learning algorithm with substructure. Among the algorithms that do not use substructures, whether to use the BPM algorithm has little impact on transfer performance.

**FIGURE 1:**
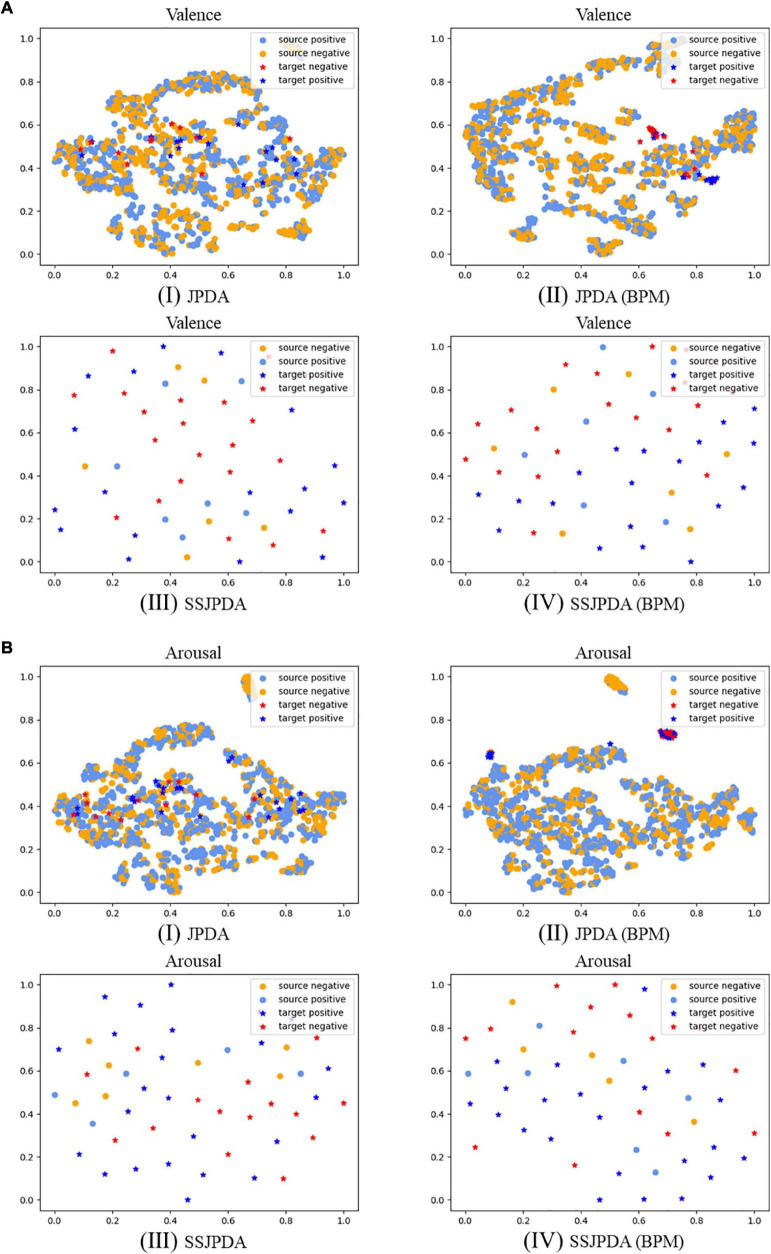
The source and target domain’s prediction samples are projected to two-dimensional visualization through t-SNE in multimodal data experiments with different algorithms. **(A)** Shows valence classification representations, and **(B)** shows arousal classification representations, where (I) is JPDA algorithm, (II) is JPDA (BPM) algorithm, (III) is SSJPDA algorithm, (IV) is SSJPDA(BPM) algorithm.

In order to present the representations generated by different methods more intuitively, we use the t-SNE algorithm in multimodal data experiments to reduce the dimension and visualize the representations generated by different algorithms. Figure 1 is the t-SNE diagrams of each algorithm in Experiment 1 on multimodal data. The dots legend represents the source domain data, and the legend of the star represents the target domain data. The light blue and dark blue represent positive samples, and the orange and red represent negative samples.

According to Figure 1, the representations generated by different algorithms have consistent performance, regardless of valence or arousal classification. The substructures generated by SSJPDA and SSJPDA-BPM through clustering in the domain can significantly reduce the quantity of data. JPDA-BPM and SSJPDA-BPM can lessen the intra-class sample distance and increase the inter-class sample distance in the same domain. At the same time, they can make the same kind of samples in different domains align better compared with not using the BPM algorithm. The representation generated by SSJPDA-BPM has better separability than others.

### Experiment 2

The source domain data and target domain data settings of Experiment 2 are the same as Experiment 1, but only fusion data is used for comparison in the different algorithms. Traditional machine learning and transfer learning algorithms are used to classify valence and arousal. Because the TCA, JDA, BDA, and JPDA algorithms all use the 1-Nearest Neighbor (1NN) model in classification, we choose 1NN as the traditional machine learning model to compare the impact of the transfer learning algorithm on recognition results. Table 3 shows the average accuracy and F1-measure of 32 subjects using different algorithms in valence and arousal.

**TABLE 3:** The average accuracy and F1-measure of different algorithms in valence and arousal classification.

Method	Valence		Arousal	
	ACC	F1	ACC	F1
1NN	0.484	0.529	0.504	0.555
TCA	0.49	0.533	0.521	0.583
JDA	0.496	0.535	0.515	0.578
BDA	0.519	0.56	0.538	0.572
JPDA	0.541	0.576	0.568	0.626
JPDA-BPM	0.533	0.615	0.573	0.613
SSJPDA	0.617	0.627	0.635	0.643
SSJPDA-BPM	**0.636**	**0.653**	**0.644**	**0.679**

The numbers in bold indicate the highest value of the experimental results.

Table 3 shows that in the problem of emotion recognition based on the DEAP dataset, when the data distribution of the source domain and target domain is different, the performance of all transfer learning algorithms is better than the 1NN algorithm. In recognition of valence and arousal, the algorithm with the worst classification accuracy in the transfer learning algorithm is still 1.2% (TCA) and 2.2% (JDA) higher than 1NN, respectively. We proposed SSJPDA-BPM algorithm has the best performance. The recognition accuracy and F1-measure values of valence are 63.3 and 65.3%, respectively. The recognition accuracy and F1-measure arousal values are 64.4 and 67.9%, respectively. Its accuracy and F1-measure values are higher than other algorithms. Compared with the traditional transfer learning algorithm, SSJPDA-BPM has higher classification accuracy than TCA, JDA, and BDA by 29.8, 28.2, and 22.5%, respectively, in valence classification. In the recognition accuracy of arousal, SSJPDA-BPM is 23.6, 25.1, and 19.7% higher than TCA, JDA, and BDA, respectively. The comparison results of whether to use BPM and SS algorithms have been described in detail in Experiment 1, which will not be explained in this part.

**FIGURE 2:**
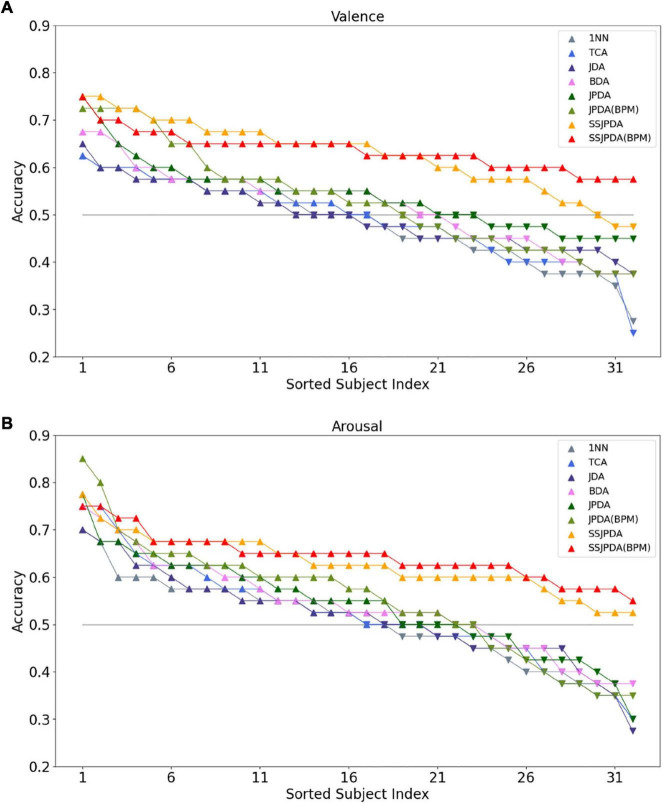
The recognition accuracy of each algorithm in Experiment 2 in 32 subjects was ranked in descending order. **(A)** Shows the recognition accuracy of valence in different algorithms of 32 subjects, and **(B)** shows the recognition accuracy of arousal in different algorithms of 32 subjects.

Figure 2 is the line chart showing the recognition accuracy of each algorithm in Experiment 2 in 32 subjects in descending order, of which Figure 2A is the recognition accuracy of valence and Figure 2B is the recognition accuracy of arousal. The gray horizontal line is the chance level of 50% for the two classes. Each color corresponds to an algorithm. Subjects above the gray level line are represented by upward triangles. The recognition accuracy of this subject in the algorithm is higher than that of the chance level. Downward triangles represent subjects below the gray level line, and the recognition accuracy of this subject in the algorithm is lower than the accuracy of the chance level.

Figure 2A shows that more than half of the subjects have a recognition accuracy higher than the chance level of 50% for two classes in recognition of valence by the 1NN, TCA, and JDA algorithms. The recognition accuracy of 1NN and TCA in some subjects is less than 30%. Therefore, the average recognition accuracy of these two algorithms is lower than JDA. By comparing JDA, BDA, and JPDA algorithms in order of this arrangement, we can see that the number of people whose three algorithms are higher than the chance level of 50% is slowly increasing. Meanwhile, the highest and lowest recognition accuracy of subjects in the test set is also gradually increasing. The performance of JPDA-BPM is lower than that of JPDA. Although JPDA-BPM algorithm has more subjects with recognition accuracy higher than 70 and 60%, wrong matching still leads to more subjects with recognition accuracy lower than 45%. The SSJPDA and SSJPDA-BPM algorithms have improved compared to the original algorithm. It is worth noting that the recognition accuracy of the SSJPDA-BPM algorithm is above 55% in all subjects.

Figure 2B shows that the number of subjects with arousal recognition accuracy higher than the chance level exceeded half of the total sample size. 1NN, TCA, and JDA algorithms have more than 70% recognition accuracy in some subjects. However, the recognition performance of the algorithm is poor in some subjects, and its recognition accuracy is lower than 35%, which leads to the low average recognition accuracy of these three algorithms. In the JPDA-BPM algorithm, one subject has a recognition accuracy of 85%, which is the highest among the eight algorithms. Meanwhile, its minimum recognition accuracy is 35%, and the number of people lower than the chance level of 50% is also higher than JPDA, which leads to little difference between its average recognition accuracy and JPDA. In the comparison between SSJPDA and SSJPDA-BPM, the performance of SSJPDA-BPM is generally higher than SSJPDA, and the recognition accuracy is lower than SSJPDA only in a few subjects. Comparing whether to use the SS method, SSJPDA and SSJPDA-BPM have been improved compared to the original algorithm, and the recognition accuracy of all subjects is above 50%.

## Discussion

### The performance of different algorithms with multi modal and single modal data

In the algorithm based on non-substructure, the recognition accuracy using multimodal data is less improved than that using single-mode data. In the substructure-based algorithm, multimodal data can significantly improve recognition performance. Multimodal data in JPDA, the amount of data in the source domain and target domain are very different. The source domain consists of 31 subjects, each of which contains 40 samples. The target domain is 40 samples from one subject, of which 40 samples are also divided into a validation set composed of 10 random samples and a test set consisting of 30 random samples. Therefore, there is an enormous difference in the data volume between the source domain and the target domain. When the source and the target domain are projected to the same feature space, the probability of false matching will increase, which affects transfer recognition’s accuracy.

Fusing the features of the four modes will increase the sample dimensions of the source and the target domain. The probability of sample error matching is greater than that of single-mode identification, so the performance of the non-substructure algorithm in multimodal data identification is poor. The transfer learning algorithm based on substructure can avoid error matching caused by sample dimensions increasing and data volume differences between the source and target domains. SSJPDA first generates substructures by clustering in the domain and then matches the substructures. The generation of substructures can dramatically reduce the data volume gap between the source and target domains. This can significantly reduce the probability of false matching. Therefore, the SSJPDA algorithm performs better than JPDA in single-mode emotion recognition. Without the influence of data volume, multimodal fusion data can provide more dimensional information to align the substructures of the source domain and target domains’ substructures. Therefore, using the SSJPDA algorithm to recognize multi-mode emotional data can obtain high recognition accuracy.

The application of BPM in SSJPDA can significantly improve recognition performance. Because the emotional labels of subjects in the DEAP dataset are provided by the subjects themselves, this will affect the consistency of the emotional labels of different subjects. At the same time, because the emotional stimulation of the DEAP dataset depends on multimedia clips, some subjects also have the problem of weak emotional stimulation. In this experiment, the source domain contains all the test samples of 31 subjects, so there must be many abnormal samples and noise in the source domain. If no substructure is generated in the source domain and the data is projected directly, the abnormal samples and noise greatly impact the projecting matrix. Therefore, the advantages of the BPM algorithm are not reflected in JPDA. However, the substructure algorithm can cluster the noise or outliers of samples into the substructures of adjacent samples to reduce the impact of noise and outliers. When the source and target domain samples are clustered into substructures, we fully consider the distribution differences between the source and target domain substructures. Projecting the substructure through two different projecting matrices can better project the substructure of the source domain and the target domain to the feature space to improve the algorithm’s recognition performance.

### The comparison of different algorithms

When the data distribution of the training set and test set is inconsistent, the traditional machine learning algorithm cannot be competent for classification. Therefore, the 1-NearestNeighbor (1NN) algorithm performs worst in this emotion recognition problem. The purpose of transfer learning is to solve the inconsistency between the data distribution of the training set and test set, that is, the inconsistency between the distribution of the source domain and target domain. Therefore, the transfer learning algorithm performs well in this emotion recognition problem. Among them, transfer component analysis (TCA) assumes that if the marginal distributions of the source domain and the target domain are close, the conditional distributions of the two domains will also be close. Therefore, TCA projects the source and target domain data together into a high-dimensional reproducing kernel Hilbert space. In this space, the data distance between the source and the target is minimized, while their respective internal attributes are preserved to the greatest extent to complete the transfer learning. The joint distribution adaptation (JDA) method simultaneously assumes that the marginal and conditional distribution of the source and target domains are different. Then the two distributions are adapted together to achieve transfer. The goal of JDA is to reduce the distance between the source and target domain’s joint probability distribution to complete the transfer learning. Balanced distribution adaptation (BDA) is improved on the basis of JDA. BDA assumes that marginal distribution adaptation and conditional distribution adaptation are not equally important. BDA adaptively adjusts the importance of marginal and conditional distribution in the distribution adaptation process according to specific data fields to complete the transfer.

We proposed the SSJPDA algorithm can better measure the distribution difference between the two domains through the joint probability distribution. This is better than JDA and BDA algorithms, which directly calculate the sum of marginal probability and conditional probability distribution differences between the two domains. In the SSJPDA algorithm, the algorithm’s transferability is achieved by minimizing the difference in joint probability distribution between different domains of the same class, and the algorithm’s discriminability is achieved by maximizing the difference in joint probability distribution between different domains. At the same time, using substructures reduces the difference in data volume between the source domain and the target domain and reduces the impact of noise or outliers. After using the substructure, the SSJPDA-BPM algorithm we proposed fully considers the distribution difference between the substructure of the source domain and the target domain and projects the substructure through two different mapping matrices to improve the performance of the algorithm further. Therefore, this paper’s SSJPDA (BMP) algorithm has the highest recognition performance accuracy.

### Discussion on negative transfer

Negative transfer means that the knowledge learned in the source domain has a negative effect on the learning in the target domain. When the source domain data is not similar to the target domain data, or the source domain data is similar to the target domain data, but the transfer learning method is not good enough that no transferable components are found, the negative transfer is likely to occur in those two cases (Pan and Yang, 2009). In this experiment, the distribution of source domain data and target domain data are different. Through the multi-source domain transfer method, the data in the target domain is correctly classified by using the knowledge learned from multiple source domains so that the target domain can learn more comprehensive feature information. This can well avoid the negative transfer caused by the low correlation between the source domain and the target domain in the single source domain transfer.

However, if the source domain data used in the transfer learning algorithm contains a lot of noise, it is likely to negatively impact the classification model. The multiple source domain transfer method will further amplify the impact of noise. Regrettably, the four physiological signals, especially EEG signals, in this experiment contain numerous noise and abnormal samples. Therefore, the noise and abnormal samples in the source and target domains will inevitably lead to negative transfer. Therefore, in addition to SSJPDA-BPM algorithm, the classification accuracy of every algorithm in some subjects is lower than the chance level of 50% for two classes.

Compared with other algorithms, SSJPDA and SSJPDA-BPM generate substructures in the source domain and target domain. These substructures can properly process the data according to the data’s similarity, which can validly reduce the negative impact of noise and abnormal samples in the source and target domains. It can effectively avoid negative transfer and improve the performance of the transfer learning algorithm. At the same time, as traditional migration learning methods, TCA, JDA, and BDA algorithms have a better effect on the transfer of feature size within a certain threshold. The information redundancy caused by too large feature vectors makes the impact of confusing information greater than that of task-related information, resulting in negative transfer (Zhang et al., 2020). However, SSJPDA and SSJPDA-BPM can filter abnormal samples affected by confusing information through substructure, which further improves the algorithm’s performance.

More than that, how to transfer the components found in the source and target domain data also affects the negative transfer. In comparing whether to use the BPM algorithm, if the algorithm finds the correct transferable components, projecting the effective data to the feature space through two different projecting matrices can improve the algorithm’s performance and better avoid the negative transfer. However, suppose there is a lot of noise and outliers in the data. In that case, the BPM algorithm changes from an excellent method that avoids more negative transfers to a lousy method that leads to more negative transfers.

## Conclusion

This paper proposes SSJPDA and SSJPDA-BPM algorithms to use the labeled physiological data to recognize the emotion of new subjects. We also explored single-mode and multimodal data’s influence on emotion recognition based on physiological signals. The performance of the SSJPDA-BPM algorithm is verified by the comparative experiments of various algorithms on DEAP dataset. The results show that SSJPDA and SSJPDA-BPM algorithms can better deal with noise and outliers in data by clustering substructures. Meanwhile, these algorithms can reduce the quantity of data that better use the multi-dimensional information provided by multimodal fusion data. BPM algorithm can project the substructure through two different projecting matrices, which can better project the source domain and target domain data to the feature space, to improve the algorithm’s recognition performance. The experimental results show that the average recognition accuracy of the proposed SSJPDA-BPM algorithm in the multimodal fusion physiological data is 63.6 and 64.4% in valence and arousal, respectively.

## Data availability statement

Publicly available datasets were analyzed in this study. This data can be found here: http://www.eecs.qmul.ac.uk/mmv/datasets/deap/.

## Author contributions

ZF, XH, and JH: conceptualization and supervision. BZ, YL, and HW: data curation. ZF, XH, and BZ: methodology, writing – original draft, and review and editing. BZ, HW, and ZF: validation. XH and YL: visualization. All authors contributed to the article and approved the submitted version.
